# Assessing spatial accessibility of community pharmacies in England and Wales using floating catchment area techniques

**DOI:** 10.1080/20523211.2025.2466203

**Published:** 2025-02-25

**Authors:** Stephen D. Clark, Andy Newing

**Affiliations:** a School of Geography and Consumer Data Research Centre, University of Leeds, Leeds, UK; b School of Geography, University of Leeds, Leeds, UK

**Keywords:** Pharmacies, accessibility, floating catchments, England and Wales

## Abstract

**Background:**

Community pharmacies in England and Wales are taking on a broader range of primary care responsibilities in order to ease pressure on other health services. ‘Pharmacy First’, launched in 2024, allows patients to access treatment for a range of common conditions directly from a pharmacy without the need to consult a GP. However, funding and workforce pressures have resulted in a number of pharmacy closures in recent years. This study assesses the geographical accessibility of community pharmacies in England and Wales and identifies the impact of these recent closures.

**Methods:**

Using open data on pharmacy locations and opening hours this study calculates a Spatial Accessibility Index (SPAI) for access to pharmacies by car in 2022 and 2024. We use a Modified Huff Variable Three Step Floating Catchment Area (MHV3SFCA), a variant of the Floating Catchment Area (FCA) technique.

**Results:**

Suburban and rural neighbourhoods tend to have poorer access to community pharmacies, whilst more deprived neighbourhoods generally have comparatively better access. We identify neighbourhoods which could be classed as ‘pharmacy deserts’, which are primarily located in rural areas. We identify that all neighbourhood area types witness a reduction in overall accessibility to community pharmacies between 2022 and 2024. In total these result in a 10% reduction in the SPAI.

**Conclusion:**

The MHV3SFCA applied here is novel in its application to community pharmacy accessibility in a UK context. We demonstrate its utility as a tool to identify the impact of changes to the community pharmacy network on accessibility as experienced by different neighbourhoods. We find evidence of a ‘positive pharmacy care law’ and also the existence of some ‘pharmacy deserts’.

## Background

Pressure has been mounting on the National Health Service (NHS) in the United Kingdom (UK), with patients struggling to obtain appointments to see general practitioner (GP) family doctors (O'Dowd, 2023) and experiencing long waiting lists for hospital treatments (Hoddinott, 2023). In response, the government and NHS strengthened the role of community pharmacies. Pharmacies demonstrated their utility during the COVID-19 pandemic, playing a key part in the rapid roll-out of vaccinations. There is evidence that the profession is keen to expand its role in community health care, fully utilising the clinical skills of pharmacists (Gibson et al., 2024; Jacobs et al., 2018).

As noted in 2024 by the governmental House of Commons Health and Social Care Committee (2024), prescribing medication – typically obtained from a community pharmacy – is the most common way of treating patients in the NHS. In Great Britain, community pharmacies are operated by a mix of large national operators (including Boots, Superdrug, Tesco and ASDA), regional chains (e.g. Cohens) and local independent operators. All pharmacies are expected to provide ‘Essential’ dispensing and advice services (Anderson & Sharma, 2020), with many pharmacies receiving additional funding to provide ‘Locally Commissioned’, ‘Advanced’ or ‘Enhanced’ services. These may include smoking cessation, sexual health advice, treatments for minor ailments, vaccinations and medication reviews (Brown et al., 2016; Latif et al., 2020). Pharmacies generate additional income from retail activities (including selling over-the-counter medication) and providing private services.

Since January 2024, many community pharmacies in England have increased their scope of practice under the ‘Pharmacy First’ scheme. They act as a single point of contact with the primary care system, assessing and treating seven common illnesses without the need for the patient to see their GP (NHS England, 2024). Pharmacies in Wales are also encouraged through additional payments to offer services beyond the types considered essential in England (Lewis, 2023). Since these additional tiers are optional, some locations may not have all these services available, with smaller pharmacies, typically located in rural areas tending to offer a more limited range of ‘essential’ only services (Merks et al., 2016).

Many rural pharmacies are supported by subsidies under the ‘Pharmacy Access Scheme’, designed to maintain patient access to ‘isolated’ pharmacies, based on their distance from the next nearest pharmacy and their prescribing volume. Following amendments to the scheme in 2022, the basic criteria for inclusion apply to a distance threshold of 1 mile (0.8 miles in the most deprived neighbourhoods) (Department of Health and Social Care, 2023).

As well as community pharmacies, distance selling pharmacies (DSPs) (Long et al., 2022) including ‘Pharmacy 2U and ‘LloydsDirect’ are NHS-registered providers that dispense medication prescribed by GPs directly to patients by post. Many community pharmacies offer home delivery of medications directly to patients, especially those who would struggle to reach their local pharmacy (e.g. due to mobility issues).

Within the wider literature, localities where populations have limited access to health services are described as ‘deserts’ (Flinterman et al., 2023), with the term pharmacy desert well-used in a North American context (e.g. see Ying et al., 2022) and beginning to be used in the UK.

In 2023, the Department of Health and Social Care (2023) reported that “access to community pharmacies is generally good with 80% of people living within 20 min walking distance of a community pharmacy and twice as many pharmacies in more deprived areas”. Whilst many forms of healthcare tend to exhibit an ‘inverse care law’ whereby the availability of healthcare is inversely related to needs (Tudor Hart, 1971), there has been evidence of a ‘positive pharmacy care law’ (Todd et al., 2014). The availability of pharmacies is comparatively better in more deprived neighbourhoods (Ministry of Housing Communities and Local Government, 2019).

In response to a recent consultation by the Health and Social Care Committee (2024), community pharmacies report a challenging operating environment as a result of growing demand, increasing costs, recruitment challenges and medicine shortages. A failure of funding to match these challenges has seen recent reductions in the number of pharmacies operating (see Table 1), particularly in rural and deprived locations (Cross et al., 2024). In the year April 2021 to March 2022 there were around 11,500 pharmacies in England, the lowest number since 2015/16 (Balogun, 2023), and further reductions by 222 between December 2022 and June 2023. The Company Chemists’ Association (2022) note that 41% of the closures between 2015 and 2022 were in the most deprived areas in England.

**Table 1.** Count of pharmacies by opening hours and total opening hours, mid-2022 and early-2024.

Measure (opening hours per week)	Mid-2022	Early-2024	Change	(%)
Open for 40 or fewer hours	780	914	+134	+17
Open between 40 and 48 or fewer hours	3668	3816	+148	+4
Open between 48 and 72 or fewer hours	5580	5352	−228	−4%
Open for more than 72 hours	1479	842	−637	−43%
**Total count of pharmacies**	**11,507**	**10,924**	−**583**	−**5%**
**Total hours**	**637,963.3**	**571,907.5**	−**66,055.8**	−**10%**

As well as these permanent closures, Healthwatch (2024) reports that there were 13,863 unscheduled temporary pharmacy closures in England in 2023, accounting for over 46,000 hours lost, with higher rates of such closures in rural areas, in areas with older populations and in areas that already suffered from poor access to primary care.

The changing nature of the pharmacy network in England and Wales, and its growing role in meeting patients’ needs in relation to accessible frontline NHS services, necessitates consideration of the ease of access to its service. Merks et al. (2014) report that in 2011, 89% of UK respondents identified location as an important factor in pharmacy choice. Penchansky and Thomas (1981) describe five dimensions for access, three are aspatial, ‘Affordability’, ‘Acceptability’, and ‘Accommodation’, while two, ‘Availability’ and ‘Accessibility’, are spatial. This study is primarily concerned with the two spatial aspects of access to community pharmacies. Availability measures the capacity of the services at a location whilst accessibility additionally captures the notion of how easy it is to travel to access the service. The utility of this geographic consideration on the availability and accessibility of pharmacies is highlighted by the recent scoping study by Fernandes et al. (2022) who found that of the 48 studies they identified, 36 (75%) were concerned with accessibility, with the majority, 31 (66%) using proximity calculations whilst the remaining 16 studies (33%) investigated associations with other variables. In terms of geographic coverage, they found that 29 (60%) come from a United States of America (USA) setting, whilst just 3 (7%) were country-level studies conducted for England (Barrett & Hodgkinson, 2019; Todd et al., 2014; Todd et al., 2018). This highlights a dearth of published studies of the situation in England.

In the literature, there are various approaches to measuring accessibility. The three most common are: a practitioner-to-population ratio that measures the number of people a practitioner typically serves, e.g. GPs per patient; a catchment which counts the number of providers within a set distance or travel time, e.g. number of dentists within 10 km; or a proximity to the closest supplier, e.g. the distance to travel to the nearest hospital. Using these approaches Todd et al. (2014) estimated that nearly 90% of the English population was within a 20-min walk of a pharmacy, although the percentage varied by the urban/rural and deprived nature of the area. Each of these three approaches has its disadvantages. The ratio approach is influenced by the size of the area under consideration and assumes that people outside the area cannot access the service and people in the area cannot use a service outside of it. The catchment and proximity approaches take no account of competition for a given service, nor the available capacity within those service providers.

To overcome some of these disadvantages the concept of a Floating Catchment Area (FCA) approach has been proposed (Luo & Wang, 2003) and applied in many studies of geographical accessibility (Page et al., 2018). At its simplest, the approach works in two steps. In the first step, the available capacity for a service is divided amongst a population within a given radius, on a practitioner-to-population basis. The second step identifies all the supply points within a given radius of a population demand point (such as a neighbourhood centroid). The practitioner ratios for these supply points are summed to provide the Spatial Accessibility Index (SPAI) for the location. The calculated index increases (denoting better accessibility for a given demand point) as: the available supply increases; competition for the service decreases; and/or the travel impedance to the service decreases. From the initial FCA approach, enhancements have been proposed including the incorporation of importance weights via discrete distance bands or distance decay functions (where the weight decreases as the distance increases), variable catchment sizes and supply competition (Langford et al., 2016; Subal et al., 2021). Such FCA approaches have been applied to assess pharmacy accessibility in the USA at the county scale (Ikram et al., 2015), as part of a state (Zhou et al., 2021), and the whole continental USA (Sharareh et al., 2024). From their scoping study, Fernandes et al. (2022) advocate the use of such techniques since they “ … [are] a robust method which can improve the understanding of accessibility”. Wang and Ramroop (2018) used SPAIs in combination with indicators of clusters of ‘vulnerable’ communities to design a combined accessibility-vulnerability score for neighbourhoods. Such methods have also been used to measure accessibility for populations with specific health needs, e.g. vaccinations (Neuner et al., 2021; Zahnd et al., 2020)

This study applies an enhanced version of the FCA methodology, the Modified Huff Variable Three Step Floating Catchment Area (MHV3SFCA) (Jörg & Haldimann, 2023), in the context of the English and Welsh community pharmacy network in mid-2022. It shows how accessibility varies over space, revealing areas that are potentially ‘pharmacy deserts’ (Flinterman et al., 2023) and also examines a more recent time period, early 2024, to see how this accessibility is changing over time.

## Methods

For this study, three categories of data are required. Firstly a supply location and capacity, here this is the location and the number of weekly opening hours of community pharmacies in England and Wales (NHS Business Services Authority, 2024). We have excluded DSPs as they are not accessed by a local residential population. The analysis is based on 11507 community pharmacies in England and Wales. Secondly a location and measure of demand, this is the mid-year 2022 population estimate produced by the Office for National Statistics (2024) located at the population-weighted centroid of 2021 Census Lower Super Output Areas (LSOAs) (Office for National Statistics, 2022). In mid-2022, the population of an LSOA was estimated to be on average 1700 people in each of the 35671 LSOAs. Finally, there is a measure of travel impedance between the demand and supply locations. Historically this was a straight line distance, but in more recent studies this is represented as road distance or a mode-specific travel time. Here this is the travel time by car as calculated using the osrm package (Giraud, 2022) in the R software (R Core Team, 2023). This algorithm uses information on free flow road speeds by road type, and the presence of one-way streets, and imposes turn and parking penalties to the journey. It does not take into account periods of traffic congestion during peak hours, when speeds are lower than the free flow road speed, or delays due to traffic signals. To limit the number of calculations, only the car travel times of the 120 straight line closest LSOAs to each pharmacy are calculated (a potential catchment population of 120 * 1700 = 204,000 people).

The method used is the MHV3SFCA variant of the FCA (Jörg & Haldimann, 2023). The advantages of incorporating the Huff initial step are detailed in Subal et al. (2021) and the rationale for the inclusion of variable catchments is made in Jörg and Haldimann (2023), with the overall advantages summarised in table 3 of Hauser (2023). The first advantage is that MHV3SFCA incorporates a Gaussian distance decay function so that the attractiveness of a pharmacy location diminishes as the distance increases. For this decay function, the threshold time that would be reasonable to travel by car to a pharmacy (*d*_max_ in the literature) is set at 10 min and the weight at *d*_max_ (*f*(*d*_max_)) is set to 0.05 (for comparison the Department of Health and Social Care (2023) suggests that a longer 20 minute walk time is reasonable). Secondly, it incorporates Huff probabilities (Huff, 1963) to ensure that demand is not over-estimated. Over-estimation occurs when the ability of alternative supply locations to meet demand is ignored. Finally, it adopts a variable time bandwidth so that all demand-side origins (neighbourhoods) are able to reach at least a minimum number of pharmacies. The choice of what is a reasonable minimum number of pharmacies to consider (denoted as Q) is guided by the literature, with values up to 10 considered reasonable in a health context (Jörg & Haldimann, 2023). Here a slightly lower value of 8 is chosen, meaning that there is an expectation that people will notionally choose from at least 8 pharmacies. Having a choice of pharmacies is important given that some pharmacies may not be able to supply all prescription medicines or services (Community Pharmacy England, 2022) or may have restricted opening hours. Calculations are performed using the R code in Hauser (2023). Results of sensitivity analysis on the form of the distance decay function, the values of *d*_max_ and of *Q*, are reported using Pearson and Kendall rank correlation statistics.

The MHV3SFCA provides two measures of access, a Spatial Density Index (Step 2.5 of Hauser (2023)) that aligns with the concept of availability and an SPAI (Step 3), a more traditional measure of accessibility, which we report and map in the following section. Summary statistics for the index are presented based on a classification that captures neighbourhood area type (Office for National Statistics, 2018), its level of deprivation (Abel et al., 2016; Ministry of Housing Communities and Local Government, 2019) and its urban/rural nature (Office for National Statistics, 2016). Our SPAI represents pharmacy accessibility in mid-2022. There has been a considerable reduction in the number of pharmacies by early 2024, including well-publicised closures of large numbers of pharmacies operated by the Boots and Lloyd’s chains. To illustrate the impact of these closures on community pharmacy accessibility and the wider utility of our methodology, the accessibility calculations are re-done using the location and opening hours of pharmacies in early 2024.

## Results

Table 1 summarises the count of pharmacies in our data, grouped by opening hours. The overall pattern shows reductions in the number of pharmacies, especially those pharmacies opening more than 72 h a week. The consequence is that over this relatively short one-and-a-half-year time period, there has been a 10% reduction in pharmacy opening hours, largely driven by the cost and staffing pressures outlined above.

**Figure 1.**
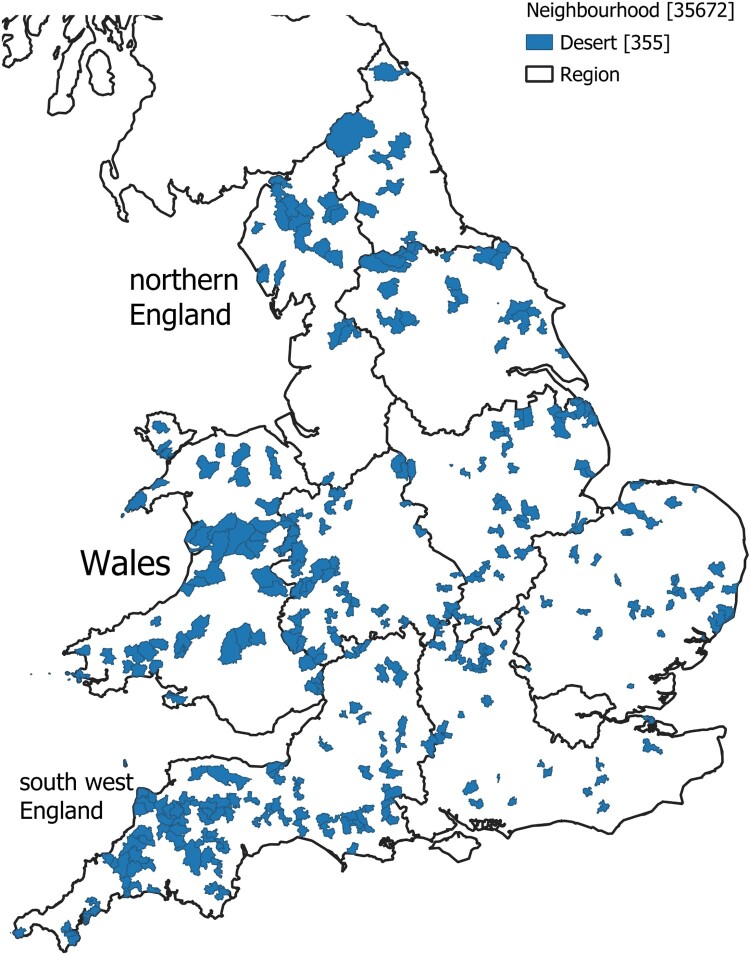
The location of potential pharmacy deserts in England and Wales.

If we define the presence of a ‘pharmacy desert’ in a relative sense by identifying the 0.1% of LSOAs nationally with the lowest SPAI, then these locations are shown in Figure 1. Immediately apparent is that there are no such deserts in the major cities and conurbations in England and Wales, and northern England appears to contain relatively few of these deserts. However, in rural parts of southwest England and along the Welsh border, there are many distinct clusters of deserts.

**Figure 2.**
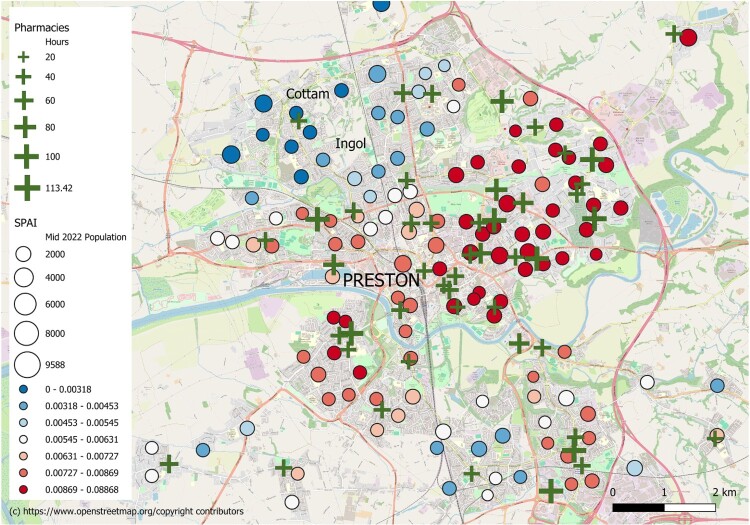
The Spatial Accessibility Index for LSOAs in Preston, England.

For a case study, the values of the SPAI for the city of Preston are shown in Figure 2. Preston is a medium-sized, northern, English town, with a rural hinterland. For context, in the 2021 Census, it has a slightly higher percentage of households without access to a car than England and Wales as a whole (27.7% vs 23.4%), but also a higher percentage who commute by car (54.0% vs 49.0%). In Figure 2, each demand point (the population-weighted centroid of an LSOA) is shown along with its corresponding SPAI score. Preston contains some neighbourhoods that score very favourably on the SPAI, including a band of neighbourhoods running from northeast to southwest which experience particularly good accessibility, with a high density of pharmacies, many with longer opening hours. However, there are also the locales of Cottam and Ingol in the northwest of the city with comparatively poor accessibility, representing some of the lowest scores identified nationally. In Cottam and Ingol, there is one pharmacy, opening for 46 hours a week, to serve a population of 9 LSOAs (∼15,000 people), and the presence of two railway lines and a motorway limits access to alternative pharmacies.

Table 2 shows the median SPAI for LSOAs of particular neighbourhood types for mid-2022 (scaled up by 1000). Firstly there is a classification of area types, with locations with younger and ethnically diverse populations having comparatively better accessibility, whilst more sub-urban and countryside populations have poorer accessibility. Secondly, there is the level of deprivation in the LSOA. Here there is a clear gradient, with the more deprived locations having better accessibility. A further gradient is also clear when looking at the urban/rural nature of the LSOA, with the more densely populated urban areas having good accessibility whilst it is very poor, by some margin, for many rural locations. Table 2 also repeats these calculations for the 2024 pharmacy network, accounting for pharmacy closures and changes in opening hours. Here there are across-the-board reductions in accessibility to pharmacies, with near double-digit reductions in the SPAI, which is in line with the observed reduction in opening hours in Table 1. The range in the percentage reduction is greatest for the area classification, whilst the percentage reductions by deprivation are mostly uniform.

**Table 2.** Median SPAI for various types in mid-2022 and early-2024 (scaled up by 1000).

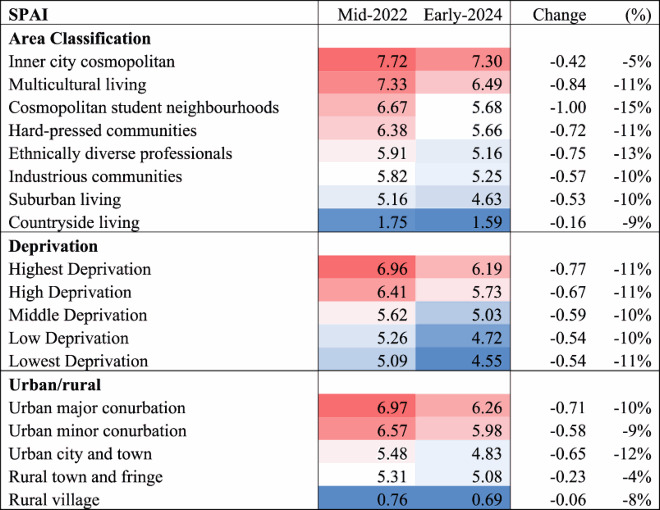

Sensitivity tests using the early-2024 situation show that the correlation between the SPAI from a Gaussian vs a linear distance decay function is high (Pearson = 0.9695; Kendall = 0.9566). Correlation for different values of Q is also high, for Q = 8 vs Q = 6 (0.9759 and 0.9888) and Q = 8 vs Q = 10 (0.9873 and 0.9946). Finally, the correlations for alternative values of *d*_max_ are a little lower, with *d*_max_ = 10 vs. *d*_max_ = 20 (0.8978 and 0.8502) and *d*_max_ = 10 vs. *d*_max_ = 30 (0.82195 and 0.74809).

## Discussion

The identification of potential pharmacy deserts using a relative threshold approach has identified both isolated and clustered LSOAs with poor access to pharmacies in some predominantly rural locations. To help improve the situation in such areas by extra or new provisions, rather than looking at individual LSOAs it might be sensible to aggregate the SPAI values to a more substantial geographic scale, either a postcode sector or a Middle Super Output Area (MSOA) (using methods outlined in Hauser (2023)).

The case study of the city of Preston illustrates how this approach can identify neighbourhoods that are underserved in terms of access to pharmacies – other cities/towns/villages could have been selected. The approach takes account of supply (the provision of a pharmacy and its count of weekly opening hours), demand (the population count of LSOAs in the neighbourhood of the pharmacy) and the travel times to alternatives. All three factors contribute to the poor accessibility in some Preston neighbourhoods, which may benefit from extended opening hours or the establishment of additional pharmacy provisions. The case study also illustrates how the clustering of pharmacy locations (Todd et al., 2018) can lead to an abundance of access in certain neighbourhoods within the city.

We find that areas with a younger population have better access to pharmacies than other age groups. Boardman et al. (2005) found that the younger age groups were more likely to buy non-prescription medicines from pharmacies whilst older people were likely to use pharmacies for the supply of prescription drugs – clearly, the balance of revenue from commercial sales versus from NHS contractual sources is a factor for pharmacy business. Another reason why age may be important is that older demographics also have a greater use of pharmacies and place value on the personal relationship with pharmacists (Wood et al., 2015). Similar to the earlier study by Todd et al. (2014) a positive care law was found to exist with people living in more deprived locations, and with assumed poorer health (Dearden et al., 2020), having better accessibility to a community pharmacy. The most dramatic difference by area type is seen for the urban/rural indicator which shows markedly reduced accessibility for rural locations, and a far higher likelihood for these locations to represent pharmacy deserts. This further exacerbates the poorer accessibility faced by rural areas in accessing many everyday services (Department of Environment, Food and Rural Affairs 2022).

The MHV3SFCA also identified twelve pharmacies (not mapped) which do not serve an obvious demand since they are not the most accessible option for any LSOAs. These are mainly located in central London, where there is an abundance of pharmacies, or in out-of-town retail centres with no population centre close by. These pharmacies may predominantly serve a workplace or leisure-based demand. Their locations may offer other advantages such as free parking, which are not captured within our modelling. A switch to using a workplace or workday-based population basis, rather than the residential basis used here, would begin to capture these uses. However such alternative counts from the 2021 Census are potentially compromised since the country was in a national COVID-19 lockdown when these counts were collected.

### Further work and limitations

Publication of the 10-year NHS plan (anticipated in spring 2025) may result in additional funding to increase pharmacy services, including the expansion of Pharmacy First. Our accessibility approach can be used to explore how extra capacity could be used to address issues in areas with poor accessibility. On the demand side, this approach can also measure the impact of population changes on accessibility.

We have not been able to capture the specific services offered at a given pharmacy, so our SPAI represents the accessibility to essential services offered by all pharmacies. Further work to capture the services offered would enable us to build a more nuanced measure of access to pharmacy services, especially Pharmacy First. It would be possible to re-do this analysis with a sub-set of the pharmacies – such as those with weekend openings – to capture access to pharmacies when many other treatment options are unavailable (see Langford et al. (2022) for an example using banking services). We could also re-run the model using specific population sub-groups (e.g. older consumers or those in poor health), who may be higher frequency users of the dispensing services offered by community pharmacies and who may face greater access barriers.

Car travel time has been used to measure travel impedance, whilst other studies have used walk time, reasoning that there are costs and availability issues associated with car ownership and public transport usage (Todd et al., 2014, 2015). Many of those studies acknowledge that walking may not be feasible, or even possible, for some sections of society. Within the osrm R package, it is possible to designate the mode of travel as walking, but it is likely that there will be some correlation between drive time and walk time meaning that whilst absolute accessibility may vary, the relative accessibility between areas will not. For a multi-modal approach to modelling journey times, Langford et al. (2016) propose an adaptation to the FCA calculations to jointly account for car, public transport and walk journey times to health care services.

A geodemographic classification built using 2011 Census data has been used in this study, and whilst there is evidence that such geodemographic (Rees, 2011) and deprivation measures (Lloyd et al., 2023) can have a long ‘shelf-life’ a more up to date geodemographic classification, when available, would be preferred.

## Conclusion

This study calculated a SPAI for pharmacies in England and Wales in 2022, and repeated to account for pharmacy closures in 2024. The MHV3SFCA applied is novel in its application to community pharmacy accessibility in the UK. Our SPAI identifies geographical inequalities in access to community pharmacies by neighbourhood area type, with many suburban and rural areas experiencing poorer access due to the sparser provision of community pharmacies in these localities, and their generally more restricted opening hours. We identify localities classed as ‘pharmacy deserts’ and suggest that future policy should seek to address this urban-rural imbalance.

Our findings support prior research which identified a potential ‘positive pharmacy care law’, with access to pharmacies being comparatively better in more deprived neighbourhoods. Pharmacy closures between 2022 and 2024 have resulted in a decline in accessibility across all neighbourhood types, yet there is no evidence that this reduction disproportionately affected rural areas or deprived neighbourhoods. We suggest that future research should use these modelling approaches to consider the relative accessibility to pharmacies among different population sub-groups and to model further anticipated changes in the pharmacy network, including new openings, all of which can be evaluated and modelled in the MHV3SFCA framework. We find that neighbourhoods typically comprised of younger populations enjoy some of the best access to community pharmacies. The excellent geographical accessibility of community pharmacies to younger populations, coupled with schemes such as Pharmacy First could cement an important role for pharmacies in the health and wellbeing of younger populations.

## Author contributions

**Conception**: Andy Newing and Stephen D. Clark; **Analysis**: Stephen D. Clark; **First draft**: Stephen D. Clark; **Revisions**: Andy Newing and Stephen D. Clark.

## Data Availability

The data used in this study is available from the websites referenced in the article. The authors do not have re-distribution rights for these data. The code used for the study is open source and referenced in the article.

## References

[CIT0001] Abel, G. A., Barclay, M. E., & Payne, R. A. (2016). Adjusted indices of multiple deprivation to enable comparisons within and between constituent countries of the UK including an illustration using mortality rates. *BMJ Open*, *6*(11), e012750. 10.1136/bmjopen-2016-012750 PMC5128942 27852716

[CIT0002] Anderson, C., & Sharma, R. (2020). Primary health care policy and vision for community pharmacy and pharmacists in England. *Pharmacy Practice (Granada)*, *18*(1), 1. 10.18549/PharmPract.2020.1.1870 PMC7092710 32256901

[CIT0003] Balogun, B. (2023). *Community pharmacy in England*. https://commonslibrary.parliament.uk/research-briefings/cbp-9854/.

[CIT0004] Barrett, R., & Hodgkinson, J. (2019). Quality evaluation of community pharmacy blood pressure (BP) screening services: An English cross-sectional survey with geospatial analysis. *BMJ Open*, *9*(12), e032342. 10.1136/bmjopen-2019-032342 PMC6924779 31831543

[CIT0005] Boardman, H., Lewis, M., Croft, P., Trinder, P., & Rajaratnam, G. (2005). Use of community pharmacies: A population-based survey. *Journal of Public Health*, *27*(3), 254–262. 10.1093/pubmed/fdi032 15870098

[CIT0006] Brown, T. J., Todd, A., O'Malley, C., Moore, H. J., Husband, A. K., Bambra, C., … Smith, S. (2016). Community pharmacy-delivered interventions for public health priorities: A systematic review of interventions for alcohol reduction,: smoking cessation and weight management, including meta-analysis for smoking cessation. *BMJ Open*, *6*(2), e009828. 10.1136/bmjopen-2015-009828 PMC4780058 26928025

[CIT0007] Community Pharmacy England. (2022). Medicine Shortages. https://cpe.org.uk/dispensing-and-supply/supply-chain/medicine-shortages/.

[CIT0008] Company of Chemists’ Association. (2022). *The impact of pharmacy closures on health inequalities*. https://thecca.org.uk/wp-content/uploads/2022/10/The-impact-of-pharmacy-closures-on-health-inequalities.pdf.

[CIT0010] Cross, R., McDonagh, S. T. J., Cockcroft, E., Turner, M., Isom, M., Lambourn, R., … Clark, C. E. (2024). Recruitment and retention of staff in rural dispensing primary care practice: A qualitative inquiry. *BJGP Open*, *8*(1), 1. 10.3399/BJGPO.2023.0130 PMC11169991 37977659

[CIT0011] Dearden, E. K., Lloyd, C. D., & Green, M. (2020). Exploring the histories of health and deprivation in Britain, 1971-2011. *Health & Place*, *61*, 102255. 10.1016/j.healthplace.2019.102255 31780387

[CIT0012] Department of Environment, Food and Rural Affairs. (2022). Overall measure of accessibility of services – 2019. https://www.gov.uk/government/statistics/rural-transport-travel-and-accessibility-statistics/overall-measure-of-accessibility-of-services-2019–2.

[CIT0013] Department of Health and Social Care. (2023). Written evidence submitted by the Department of Health and Social Care (PHA0018) in response to the Health and Social Care Committee inquiry into Pharmacy Services.

[CIT0014] Fernandes, B. D., Foppa, A. A., Almeida, P. H. R. F., Lakhani, A., & de Mendonça Lima, T. (2022). Application and utility of geographic information systems in pharmacy specific health research: A scoping review. *Research in Social and Administrative Pharmacy*, *18*(8), 3263–3271. 10.1016/j.sapharm.2021.11.004 34836813

[CIT0016] Flinterman, L. E., González-González, A. I., Seils, L., Bes, J., Ballester, M., Bañeres, J., … Likic, R. (2023). Characteristics of medical deserts and approaches to mitigate their health workforce issues: A scoping review of empirical studies in western countries. *International Journal of Health Policy and Management*, *12*(1), 1–16. 10.34172/ijhpm.2023.7454 PMC10590222 38618823

[CIT0017] Gibson, H., Sanders, C., Blakeman, T., Ashcroft, D. M., Fudge, N., & Howells, K. (2024). Providing care to marginalised communities: A qualitative study of community pharmacy teams. *British Journal of General Practice*, *74*(738), e49–e55. 10.3399/BJGP.2023.0267 PMC10755997 38154937

[CIT0018] Giraud, T. (2022). Osrm: Interface between R and the OpenStreetMap-based routing service OSRM. *Journal of Open Source Software*, *7*(78), 78. 10.21105/joss.04574

[CIT0019] Hauser, J. (2023). *Spatial accessibility to paediatricians in Switzerland. An improved application approach of the MHV3SFCA method*. University of Zurich. https://www.zora.uzh.ch/id/eprint/252654/1/Thesis_Hauser.pdf.

[CIT0020] Health and Social Care Committee. (2024). *Pharmacy: House of Commons Committee HC140 – Third report of session 2023–24*. House of Commons.

[CIT0021] Healthwatch. (2024). Pharmacy closures in England. Newcastle: Healthwatch England. https://www.healthwatch.co.uk/report/2024-09-26/pharmacy-closures-england.

[CIT0022] Hoddinott, S. (2023). The NHS crisis. Institute for Government. https://www.instituteforgovernment.org.uk/publication/nhs-crisis.

[CIT0023] Huff, D. L. (1963). A probabilistic analysis of shopping center trade areas. *Land Economics*, *39*(1), 81–90. 10.2307/3144521

[CIT0024] Ikram, S. Z., Hu, Y., & Wang, F. (2015). Disparities in spatial accessibility of pharmacies in Baton Rouge,: Louisiana. *Geographical Review*, *105*(4), 492–510. doi:10.1111/j.1931-0846.2015.12087.x

[CIT0025] Jacobs, S., Fegan, T., Bradley, F., Halsall, D., Hann, M., & Schafheutle, E. I. (2018). How do organisational configuration and context influence the quantity and quality of NHS services provided by English community pharmacies? A qualitative investigation. *PLoS One*, *13*(9), e0204304. 10.1371/journal.pone.0204304 30235289 PMC6147574

[CIT0026] Jörg, R., & Haldimann, L. (2023). Mhv3sfca: A new measure to capture the spatial accessibility of health care systems. *Health & Place*, *79*, 102974. 10.1016/j.healthplace.2023.102974 36708664

[CIT0027] Langford, M., Higgs, G., & Fry, R. (2016). Multi-modal two-step floating catchment area analysis of primary health care accessibility. *Health & Place*, *38*, 70–81. 10.1016/j.healthplace.2015.11.007 26798964

[CIT0028] Langford, M., Price, A., & Higgs, G. (2022). Combining temporal and multi-modal approaches to better measure accessibility to banking services. *ISPRS International Journal of Geo-Information*, *11*(6), 350. 10.3390/ijgi11060350

[CIT0029] Latif, A., Mandane, B., Ali, A., Ghumra, S., & Gulzar, N. (2020). A qualitative exploration to understand access to pharmacy medication reviews: Views from marginalized patient groups. *Pharmacy*, *8*(2), 73. 10.3390/pharmacy8020073 32357462 PMC7357163

[CIT0030] Lewis, R. (2023). *Community pharmacy in Scotland and Wales: Supplementary material*. The King's Fund. https://www.nuffieldtrust.org.uk/sites/default/files/2023-09/Community%20pharmacy%20in%20Scotland%20and%20Wales_WEB.pdf.

[CIT0031] Lloyd, C. D., Norman, P. D., & McLennan, D. (2023). Deprivation in England, 1971-2020. *Applied Spatial Analysis and Policy*, *16*(1), 461–484. 10.1007/s12061-022-09486-8 36405332 PMC9667433

[CIT0032] Long, C. S., Kumaran, H., Goh, K. W., Bakrin, F. S., Ming, L. C., Rehman, I. U., … Tan, C. S. (2022). Online pharmacies selling prescription drugs: Systematic review. *Pharmacy*, *10*(2), 42. 10.3390/pharmacy10020042 35448701 PMC9031186

[CIT0033] Luo, W., & Wang, F. (2003). Measures of spatial accessibility to health care in a GIS environment: Synthesis and a case study in the Chicago region. *Environment and Planning B: Planning and Design*, *30*(6), 865–884. 10.1068/b29120 34188345 PMC8238135

[CIT0034] Merks, P., Kaźmierczak, J., Olszewska, A. E., & Kołtowska-Häggström, M. (2014). Comparison of factors influencing patient choice of community pharmacy in Poland and in the UK, and identification of components of pharmaceutical care. *Patient Preference and Adherence*, *2014*(8), 715–726. 10.2147/PPA.S53829 PMC4029754 24868150

[CIT0035] Merks, P., Swieczkowski, D., & Jaguszewski, M. J. (2016). Patients’ perception of pharmaceutical services available in a community pharmacy among patients living in a rural area of the United Kingdom. *Pharmacy Practice (Granada*, *14*(3), 10.18549/PharmPract.2016.03.774 PMC5061519 27785163

[CIT0036] Ministry of Housing Communities and Local Government. (2019). English indices of deprivation 2019. https://www.gov.uk/government/statistics/english-indices-of-deprivation-2019.

[CIT0037] Neuner, J. M., Zhou, Y., Fergestrom, N., Winn, A., Pezzin, L., Laud, P. W., & Beyer, K. (2021). Pharmacy deserts and patients with breast cancer receipt of influenza vaccines. *Journal of the American Pharmacists Association*, *61*(6), e25–e31. 10.1016/j.japh.2021.07.006 34340925 PMC8783974

[CIT0038] NHS Business Services Authority. (2024). Consolidated Pharmaceutical List. https://opendata.nhsbsa.net/dataset/consolidated-pharmaceutical-list.

[CIT0040] NHS England. (2024). Pharmacy First. https://www.england.nhs.uk/primary-care/pharmacy/pharmacy-services/pharmacy-first/.

[CIT0041] O'Dowd, A. (2023). Gp patient survey: Getting an appointment is harder but decline in satisfaction slows. *BMJ*, *382*, 10.1136/bmj.p1629 37451806

[CIT0042] Office for National Statistics. (2016). 2011 rural/urban classification. https://www.ons.gov.uk/methodology/geography/geographicalproducts/ruralurbanclassifications/2011ruralurbanclassification.

[CIT0043] Office for National Statistics. (2018). 2011 area classification for super output areas. https://www.gov.uk/government/statistics/2011-area-classification-for-super-output-areas.

[CIT0044] Office for National Statistics. (2022). Census geography: An overview of the various geographies used in the production of statistics collected via the UK census. https://www.ons.gov.uk/methodology/geography/ukgeographies/censusgeography.

[CIT0045] Office for National Statistics. (2024). Population estimates by output areas, electoral, health and other geographies, England and Wales Statistical bulletins. https://www.ons.gov.uk/peoplepopulationandcommunity/populationandmigration/populationestimates/bulletins/annualsmallareapopulationestimates/previousReleases.

[CIT0046] Page, N., Langford, M., & Higgs, G. (2018). An evaluation of alternative measures of accessibility for investigating potential ‘deprivation amplification’ in service provision. *Applied Geography*, *95*, 19–33. 10.1016/j.apgeog.2018.04.003

[CIT0047] Penchansky, R., & Thomas, J. W. (1981). The concept of access: Definition and relationship to consumer satisfaction. *Medical Care*, *19*(2), 127–140. 10.1097/00005650-198102000-00001 7206846

[CIT0048] R Core Team. (2023). *R: A language and environment for statistical computing*. R Foundation for Statistical Computing. https://www.R-project.org/.

[CIT0049] Rees, P. (2011). The dynamics of populations large and small: Processes, models and futures. In J. Stillwell, & M. Clarke (Eds.), *Population dynamics and projection methods*. Springer. 10.1007/978-90-481-8930-4_1

[CIT0050] Sharareh, N., Zheutlin, A. R., Qato, D. M., Guadamuz, J., Bress, A., & Vos, R. O. (2024). Access to community pharmacies based on drive time and by rurality across the contiguous United States. *Journal of the American Pharmacists Association*, *64*(2), 476–482. 10.1016/j.japh.2024.01.004 38215823

[CIT0051] Subal, J., Paal, P., & Krisp, J. M. (2021). Quantifying spatial accessibility of general practitioners by applying a modified huff three-step floating catchment area (MH3SFCA) method. *International Journal of Health Geographics*, *20*(1), 1–14. 10.1186/s12942-021-00263-3 33596931 PMC7888693

[CIT0052] Todd, A., Copeland, A., Husband, A., Kasim, A., & Bambra, C. (2014). The positive pharmacy care law: An area-level analysis of the relationship between community pharmacy distribution,: urbanity and social deprivation in England. *BMJ Open*, *4*(8), e005764. 10.1136/bmjopen-2014-005764 PMC4156797 25116456

[CIT0053] Todd, A., Copeland, A., Husband, A., Kasim, A., & Bambra, C. (2015). Access all areas? An area-level analysis of accessibility to general practice and community pharmacy services in England by urbanity and social deprivation. *BMJ Open*, *5*(5), e007328. 10.1136/bmjopen-2014-007328 PMC4431167 25956762

[CIT0054] Todd, A., Thomson, K., Kasim, A., & Bambra, C. (2018). Cutting care clusters: The creation of an inverse pharmacy care law? An area-level analysis exploring the clustering of community pharmacies in England. *BMJ Open*, *8*(7), e022109. 10.1136/bmjopen-2018-022109 PMC6074640 30068619

[CIT0055] Tudor Hart, J. (1971). The inverse care Law. *The Lancet*, *297*(7696), 405–412. 10.1016/S0140-6736(71)92410-X 4100731

[CIT0056] Wang, L., & Ramroop, S. (2018). Geographic disparities in accessing community pharmacies among vulnerable populations in the greater Toronto area. *Canadian Journal of Public Health*, *109*(5-6), 821–832. 10.17269/s41997-018-0110-1 30073553 PMC6964368

[CIT0057] Wood, K., Gibson, F., Radley, A., & Williams, B. (2015). Pharmaceutical care of older people: What do older people want from community pharmacy? *International Journal of Pharmacy Practice*, *23*(2), 121–130. 10.1111/ijpp.12127 24905628

[CIT0058] Ying, X., Kahn, P., & Mathis, W. S. (2022). Pharmacy deserts: More than where pharmacies are. *Journal of the American Pharmacists Association*, *62*(6), 1875–1879. 10.1016/j.japh.2022.06.016 35953379

[CIT0060] Zahnd, W. E., Harrison, S. E., Stephens, H. C., Messersmith, A. R., Brandt, H. M., Hastings, T. J., & Eberth, J. M. (2020). Expanding access to HPV vaccination in South Carolina through community pharmacies: A geospatial analysis. *Journal of the American Pharmacists Association*, *60*(6), e153–e157. 10.1016/j.japh.2020.05.005 32580908

[CIT0061] Zhou, Y., Beyer, K. M. M., Laud, P. W., Winn, A. N., Pezzin, L. E., Nattinger, A. B., & Neuner, J. (2021). An adapted two-step floating catchment area method accounting for urban-rural differences in spatial access to pharmacies. *Journal of Pharmaceutical Health Services Research*, *12*(1), 69–77. 10.1093/jphsr/rmaa022 33717229 PMC7938828

